# Yeast as a tool for studying proteins of the Bcl-2 family

**DOI:** 10.15698/mic2015.03.193

**Published:** 2015-03-02

**Authors:** Peter Polčic, Petra Jaká, Marek Mentel

**Affiliations:** 1 Department of Biochemistry, Faculty of Natural Sciences, Comenius University, Bratislava, Slovak Republic.

**Keywords:** yeast, Saccharomyces cerevisiae, Bcl-2 family, Bax, Bcl-XL, BH3-only

## Abstract

Permeabilization of the outer mitochondrial membrane that leads to the release of cytochrome *c* and several other apoptogenic proteins from mitochondria into cytosol represents a commitment point of apoptotic pathway in mammalian cells. This crucial event is governed by proteins of the Bcl-2 family. Molecular mechanisms, by which Bcl-2 family proteins permeabilize mitochondrial membrane, remain under dispute. Although yeast does not have apparent homologues of these proteins, when mammalian members of Bcl-2 family are expressed in yeast, they retain their activity, making yeast an attractive model system, in which to study their action. This review focuses on using yeast expressing mammalian proteins of the Bcl-2 family as a tool to investigate mechanisms, by which these proteins permeabilize mitochondrial membranes, mechanisms, by which pro- and antiapoptotic members of this family interact, and involvement of other cellular components in the regulation of programmed cell death by Bcl-2 family proteins.

## INTRODUCTION

Proteins of the Bcl-2 family are recognized as principal regulators of programmed cell death in mammalian cells 1. They regulate the release of the cytochrome *c* and several other apoptogenic proteins from mitochondria in response to a broad range of various death-inducing stimuli, which constitutes a commitment point of mitochondrial apoptotic pathway. When released into cytosol, cytochrome *c* binds to the cytosolic protein APAF-1 and induces its oligomerization into a complex called apoptosome that activates caspases. Mechanisms, by which proteins of the Bcl-2 family operate to permeabilize mitochondrial membranes, still remain unclear. This review focuses on the use of yeast as a model to study their action.

**Figure 1:**
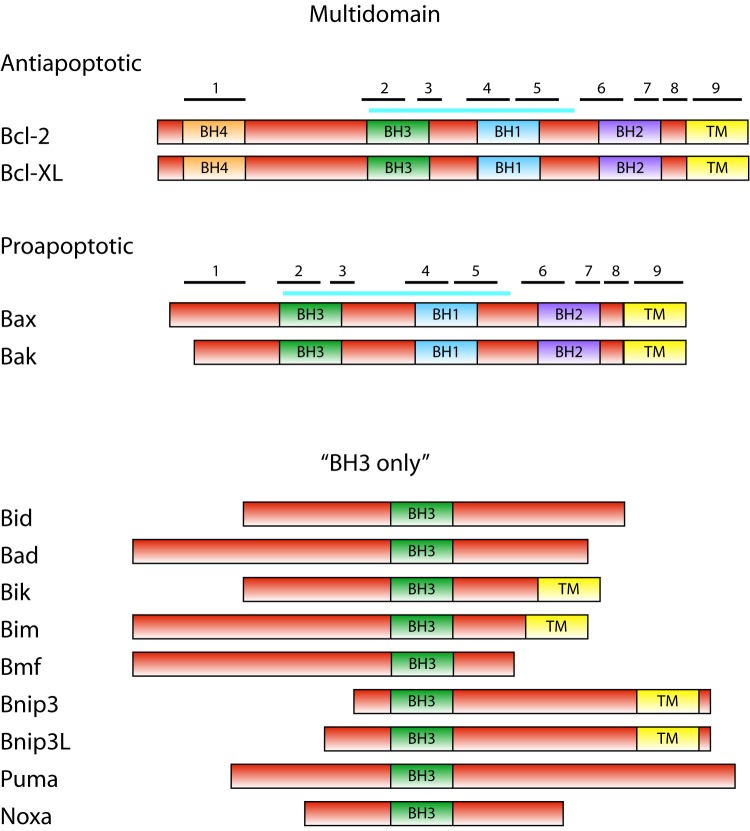
FIGURE 1: The Bcl-2 family. Domain structure of Bcl-2 family proteins is shown schematically with sizes of proteins roughly in scale. Position of BH domains is indicated. In multidomain anti- and proapoptotic proteins, positions of α-helices are indicated with numbered black bars. Blue bar indicates the position of the hydrophobic groove. Members of the Bcl-2 family not studied in yeast and not mentioned in text are omitted.

Bcl-2 family consists of proteins that share a homology with the founding member, Bcl-2 (B-cell lymphoma), in at least one of four conserved domains called BH (Bcl-2 homology) domains 2. Presence of these domains in particular Bcl-2 family member proteins correlates with their function. Based on both presence of BH domains and the function, Bcl-2 proteins can be classified into three subfamilies (Figure 1).

The subfamily of antiapoptotic proteins consists of Bcl-2 family members, e.g. Bcl-XL and Bcl-2, which contain all four BH domains. These proteins are located either in the outer mitochondrial membrane (Bcl-XL) or in all intracellular membranes (Bcl-2), and they inhibit the activity of proapoptotic proteins in living cells. The proapoptotic proteins Bax and Bak, which contain three BH domains (BH1-BH3), constitute the second subfamily. These proteins are essential for the permeabilization of the mitochondrial membrane and the release of the cytochrome *c* from mitochondria. In the absence of death signal, monomeric Bax and Bak are located in the cytosol or outer mitochondrial membrane, respectively. The third subfamily of Bcl-2 proteins consists of BH3-only proteins. These are proapoptotic members of the Bcl-2 family that contain only one of the homologous domains - BH3. Inactive BH3-only proteins exhibit diverse cellular locations in the absence of proapoptotic signal. In response to the death signal, BH3-only proteins are activated, mostly by a posttranslational modification, and translocated into the mitochondria. They induce the mitochondrial translocation of Bax, the oligomerization of Bax or Bak in mitochondrial membranes and release of the cytochrome *c* from mitochondria.

## BAX - A PORE-FORMING PROAPOPTOTIC PROTEIN

Yeast *Saccharomyces cerevisiae*, whose genome does not encode for the apparent homologues of Bcl-2 family proteins, has been considered as a model system, in which to study Bcl-2 proteins since the observation that the expression of murine Bax induces the cell death in yeast. This has been reported for the first time when the yeast two-hybrid system has been employed to study the interactions among Bcl-2 family members. Expression of hybrid molecules containing multidomain proapoptotic protein Bax appeared to be toxic for yeast and this toxicity was suppressed by the co-expression of fusion proteins containing antiapoptotic members of the Bcl-2 family 3. Expression of native murine Bax in yeast was also found to be cytotoxic and its cytotoxicity to be dependent on the homodimerization and mitochondrial localization 4. In yeast cells expressing Bax, the release of the cytochrome *c* from mitochondria was observed 5, indicating that Bax permeabilizes mitochondrial membranes, which corresponds to its activity in mammalian cells. Moreover, several markers, typical for mammalian apoptosis, including the loss of asymmetric distribution of phosphatidylserine in plasma membrane, plasma membrane blebbing, chromatin condensation and margination, and DNA fragmentation, have been observed in Bax-expressing yeast cells 6. It should be noted here that the paper reporting the latter, together with another one reporting a similar phenotype in a yeast mutant 7, laid the foundation for the field of yeast apoptosis, which has been well established by now (for review see e.g. 8).

In mitochondrial membranes, isolated from yeast cells expressing human Bax, a high conductance channel detected by patch clamping technique has been reported 9. This channel has essentially the same electrophysiological characteristics as the mitochondrial apoptosis-induced channel (MAC) that is observed in mitochondria of human fibroblasts, in which apoptosis was induced by growth factor withdrawal 9. This was the first report of a pore with the size, inferred from single-channel conductance, sufficient to let particles as large as cytochrome *c* to diffuse through the outer mitochondrial membrane. As MAC differed in channel characteristics from channels formed by purified Bax in artificial membranes 10,11,12,13, it is likely that, in addition to Bax, formation of MAC does require a component present in both yeast and mammalian mitochondria but absent in *in vitro* systems used 9.

### Bax and the permeability transition pore

In a hunt for mitochondrial components that are required for the formation of a pore by Bax, cell killing activity of Bax was tested in yeast strains devoid of selected mitochondrial components due to deletions of corresponding genes. Among these, a lot of attention was paid to putative components of the mitochondrial permeability transition pore (PTP) - a large nonselective pore in mitochondrial membranes implicated in several forms of cell death 14. These components, including the voltage-dependent anion channel (VDAC) of the outer mitochondrial membrane and the ATP/ADP carrier of the inner membrane, both of which have been suggested to play a role in permeabilization of mitochondrial membranes by Bcl-2 proteins 15,16,17, as well as several others have been tested. Although it was initially reported that both the VDAC 17,18 and the ATP/ADP carrier 15 may be involved in Bax-induced killing in yeast, further experiments have shown that both of these proteins are dispensable for Bax-mediated killing of yeast cells. Yeast strains lacking VDAC due to the deletion of *POR1* gene do not differ from wild type strains in their sensitivity towards expression of Bax 19,20 and they do form MAC when Bax is expressed 9. Strains with deletions of the genes encoding for three isoforms of the ATP/ADP carrier (*AAC1*, *AAC2* and *AAC3*) are sensitive towards the action of Bax with only a slight delay in dying, which can be attributed to the lower growth rate of these strains 21,22.

The conclusion that these components and the permeability transition pore are not required for Bax activity has also been confirmed in the mammalian system. After production of a knock-out mice with impaired ability to form PTP due to the deletion of a gene encoding for mitochondrial cyclophilin D, a model of Bax acting independently of PTP has been generally accepted 23,24,25. The absence of cyclophilin D renders mitochondria of these mice strikingly less susceptible to PTP opening by Ca^2+^, and completely insensitive to cyclosporin A, which are two of the features that define PTP, while these mice do not manifest any defects in mitochondrial apoptotic pathway 23,24,25.

Recent developments in our understanding of the permeability transition pore may once again point to the possible link between Bax and PTP. In a genetic screening for yeast mutants that are resistant to the expression of Bax, the mitochondrial F_1_F_O_-ATPase was identified 26. The F_1_F_O_-ATPase was recently identified as a central constituent of the permeability transition pore in mammalian cells 27 and in yeast 28. One can, therefore, speculate that Bax may, in fact, regulate the activity of PTP, and this would not necessarily contradict the observations on cyclophilin D knock-out mice mentioned above 23,24,25. This would assume that Bax is able to convert F_1_F_O_-ATPase into PTP without participation of cyclophilin D and the resulting pore would, indeed, be cyclosporin A insensitive.

### Bax and cardiolipin

Another component of the mitochondrial membrane suggested to play a role in Bax-mediated release of the cytochrome *c* is a unique mitochondrial phospholipid - cardiolipin, which was reported to be required for binding to and permeabilization of phospholipid vesicles by Bax and BH3-only protein tBid *in vitro* 29,30. Yeast mutants with the deletion of *CRD1* (*CLS1*), gene encoding for cardiolipin synthase, are viable even if they do not contain cardiolipin in mitochondrial membranes 31. Growth of these strains on nonfermentable carbon sources, hence the proper activity of oxidative phosphorylation, however, requires phosphatidylglycerol, a biosynthetic precursor of cardiolipin, which accumulates in mitochondrial membranes in these strains and is able to functionally substitute cardiolipin in many functions 32,33. Deletion of *PGS1* (*PEL1*), the gene encoding for phosphatidylglycerolphosphate synthase, produces a strain that does not contain detectable phosphatidylglycerol and cardiolipin in mitochondrial membranes and grows poorly on nonfermentable substrate 32,34. Using *CRD1* and *PGS1* deletion yeast strains it was shown, that mitochondria lacking cardiolipin are able to release cytochrome *c* when treated with Bax 35 and that the expression of Bax in these strains induces killing as it does in wild type strains 36. Yeast experiments, however, did not address the possible involvement of cardiolipin in tBid binding to mitochondria that has been well established in other experimental systems 37,38. Experiments with yeast mitochondria lacking cardiolipin, on the other hand, indicated that cardiolipin may affect the release of cytochrome *c* from mitochondria by binding the cytochrome *c* to the inner mitochondrial membrane 35, stressing that the release of the cytochrome *c* likely occurs in two steps. First, cytochrome *c* is detached from the inner membrane and second, the outer membrane is permeabilized by Bax and the solubilized cytochrome *c* escapes from the intermembrane space.

### Other proteins interacting with Bax

In order to identify other proteins interacting with Bax, several genetic screenings have been performed. Screening of the mammalian library for the clones that suppress the cell killing activity of Bax in yeast led to the identification of BI-1 (Bax inhibitor-1), the first member of a novel class of Bax inhibitors 39, homologues of which were also identified in non-mammalian animals, plants, fungi, bacteria and viruses (see section ‘Bxi1p/Ybh3p - a yeast protein in question‘).

In another screen for Bax suppressors, a clone from murine cDNA library encoding for sphingomyelin synthase 1 (mSMS1) was identified 40. This enzyme catalyzes the synthesis of sphingomyelin by the transfer of phosphocholine from phosphatidylcholine to ceramide 41. Expression of mSMS1 would thus in yeast lead to the decrease in ceramide level and may affect the killing by Bax (see below).

Screening of the plant library identified another suppressor of Bax-induced lethality in yeast, glutathione S-transferase/peroxidase (BI-GST, Bax inhibiting-glutathione S-transferase) 42. This enzyme has also been shown to protect yeast cells from oxidative stress induced by hydrogen peroxide in the absence of Bax. Several other plant enzymes, on the other hand, identified in screens for their stress-protective activity, were also shown to suppress lethality in yeast induced by expression of Bax, e.g. phospholipid hydroperoxide glutathione peroxidase 43. These enzymes are clearly able to protect cells from oxidative stress that the expression of Bax induces downstream of the membrane permeabilization. However, as the suppression of Bax-induced lethality by expression of BI-GST appears very efficient, in fact comparable with inhibition by Bcl-2, and as the expression of BI-GST suppresses effects of Bax on mitochondrial and vacuolar morphology 44, it may not be ruled out that BI-GST actually impairs the ability of Bax to create pores in mitochondrial membranes.

Yet another protein investigated in yeast for its ability to suppress the effects of Bax is the mammalian prion protein PrP. It has been shown that the expression of PrP protects cultured human neurons against cell death induced by overexpression of Bax induced by microinjection of Bax cDNA 45. In normal cells, PrP is a GPI-anchored protein localized at the outer surface of plasma membrane 46. Several versions of the prion protein have been co-expressed with Bax in yeast. Because native human PrP does not enter the secretory pathway very effectively when expressed in yeast, a modified version that is processed properly was used and shown to be effective in suppressing Bax activity 47. A truncated version that lacks the signal sequence and is thus localized in the cytosol is not protective 47. Interestingly, expression of the native human PrP, which is mostly localized in cytosol, was reported to suppress Bax-induced lethality 48. Together these data indicate that both the cell surface-bound and the cytosolic PrP are likely capable of the inhibition of Bax. Although not using yeast, interesting extension of these studies recently indicated that pathogenic forms of PrP, that are associated with Creutzfeldt-Jakob disease and fatal familial insomnia 49 or Gerstmann-Sträussler-Scheinker syndrome 50, are defective in suppressing Bax.

### Structure-function relationships and targeting of Bax to the mitochondria

As described above, plain expression of Bax in yeast can induce cell death, indicating that expressed Bax protein does not require activation and is rather constitutively active. This may, indeed, reflect the absence of antiapoptotic proteins, or other molecules, interacting with Bax, in yeast. However, a number of papers report the expression of Bax in inactive form, which only induces cell death when activated 51,52,53. It has been suggested that the latter behavior is associated with the expression of native human Bax while constitutive activity is typical for tagged or modified Bax protein 54. There is, however, no consensus on this issue, as both modified and unmodified versions of human or murine proteins have been described to be constitutively active, e.g. 4,15,26,39,55,56.

**Figure 2:**
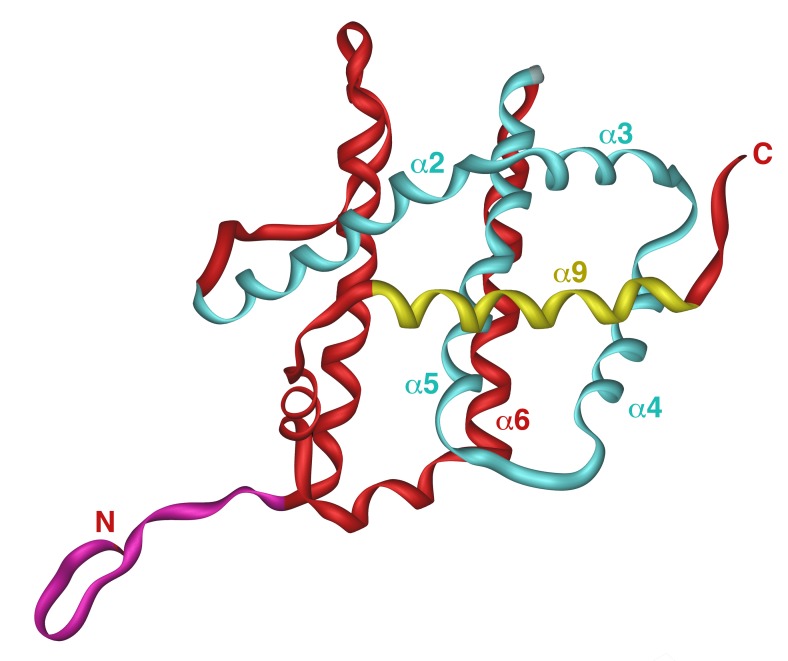
FIGURE 2: Structure of the human Bax. NMR structure of the human Bax is shown with following structural features emphasized: N-terminal domain in purple, hydrophobic groove in blue and α-helix 9 in yellow. Picture was generated by iMol 0.40 software using structural data from 57.

Yeast expression system that uses unmodified version of human Bax producing inactive protein that only kills yeast cells when activated (e.g. by co-expression of proapoptotic BH3-only protein Puma), has been employed to characterize regions and amino acid residues that are important for Bax activation and its interaction with mitochondrial membranes 54. The three-dimensional structure of soluble Bax, as determined by NMR, consists of nine α-helices connected with short loops (Figure 2) 57. Interestingly, the overall fold of proapoptotic Bax closely resembles that of antiapoptotic Bcl-XL 58. In both proteins, helices are arranged in a way that resembles translocation domains of bacterial toxins, such as diphtheria toxin and colicins. While the C-terminal helix (α9) in Bcl-XL forms a transmembrane domain that integrates Bcl-XL into the outer mitochondrial membrane, corresponding helix in Bax appears to be a mobile helix that is involved in regulation of Bax function. In soluble Bax, helix α9 is bound to a hydrophobic groove, where it likely inhibits the homodimerization and the heterodimerization of Bax with other members of the Bcl-2 family (Figure 2). When displaced from this groove, α9 was proposed to interact with the mitochondrial membrane and to regulate Bax insertion into the membrane 57. Therefore, the C-terminal helix is the one, at which a lot of attention has been focused. Expression of human Bax C-terminally tagged with c-myc epitope revealed that the addition of a tag converts cytosolic Bax into mitochondrially localized. The same effect was observed when α9 was partially replaced with a random sequence 51. Expression of Bax, in which α9 was replaced with the corresponding transmembrane helix from Bcl-XL, resulted in mitochondrially localized protein that was not able to induce cell death in yeast, as well as in transfected mammalian cells 59. These data indicate that α9 of Bax does not function as a true membrane anchor, as it is in case of α9 in Bcl-XL. The serine within α9 (position 184) is, however, likely a key residue involved in regulation of interaction of Bax with the mitochondrial membrane. Deletion of this residue converts α9 into functional membrane anchor 57. This serine has also been identified as a target for phosphorylation (in mammalian cells), which renders Bax incapable of translocation into the mitochondrial membrane 60. Other point mutations in the α9 that disrupt its interaction with hydrophobic groove or render the loop just upstream from α9 more flexible result in constitutively active protein in yeast that is both mitochondrially localized and capable of inducing cell death response 52. Taken together, these data indicate that the displacement of α9 from hydrophobic groove is crucial for activation of Bax likely by enabling the dimerization of Bax rather than providing a membrane anchor 54.

Another domain intensively studied is a domain consisting of the first nineteen residues of Bax (Figure 2). The isoforms of Bax lacking this domain have been identified in rat ischemic brain cells 61 and human glioblastoma cells 62. These isoforms, called Baxκ or Baxψ, respectively, are produced by alternative splicing of Bax mRNA, utilizing an alternative initiation codon corresponding to the methionine in position 20 of full length Bax (also called Baxα). Baxψ is more effectively translocated to the mitochondria in mammalian cells 63. Expression of N-terminally truncated Bax (corresponding to Baxψ) in yeast resulted in active protein capable to release cytochrome *c* as well as to induce death 51,63. For its ability to alter the localization of Bax that is required for its activation, this domain has been named ART (for Apoptotic Regulation of Targeting). As this domain in Bax is very mobile, it may be that the movement of ART domain regulates the Bax targeting to the mitochondria. Site directed mutagenesis introducing changes of arginine residue within ARS domain (position 9) and of aspartate residue (position 154) within BH2 domain, which likely interact electrostatically in inactive Bax, demonstrated that the disruption of the electrostatic interaction between these residues leads to mitochondrial targeting and constitutively active Bax in yeast cells 64.

Besides the role of ARS and α9 helix, involvement of the protein import machinery of the outer mitochondrial membrane (TOM complex) in targeting of Bax into mitochondrial membranes was investigated in yeast. When human Bax is expressed in a yeast mutant expressing low level of Tom22, a receptor subunit of TOM complex, decreased mitochondrial targeting 65, decreased release of cytochrome *c* 66 and physical interaction of Bax and Tom22 were reported 67. Contradicting results, on the other hand, demonstrated that Bax is integrated into yeast mitochondria independently of TOM 68. The involvement of the protein import machinery in the process of integrating of Bax into mitochondria thus remains unclear.

### Why does the expression of Bax kill yeast?

Although the yeast is used as a model to understand the activity of Bax in mammalian cells, it is, obviously, important to understand why and how yeast cells expressing Bax die. The observation that, in yeast, Bax permeabilizes mitochondrial membrane for cytochrome *c* and a significant release of cytochrome *c* from mitochondria into cytosol occurs 5, as well as the fact that the killing effect of Bax can be prevented by co-expression of antiapoptotic proteins of the Bcl-2 family 55, indicates that the mechanism, by which Bax kills yeast cells, is related to that, by which Bax acts in mammalian cells. As yeast cells do not have the homologues of mammalian downstream effectors, such as APAF-1, which are required for cell death response to the release of the cytochrome *c* in mammals, the presence of cytochrome *c* in the cytoplasm does not appear to be involved in inducing cell death in yeast. Accordingly, it has been shown that yeast mutants lacking cytochrome *c* due to the deletion of genes encoding for two isoforms of apocytochrome *c* (*CYC1* and *CYC7*) are killed by expression of Bax to the same extent as the wild type yeast 19,21.

Even though the permeabilization of mitochondrial membranes and the release of proteins from the mitochondrial intermembrane space into the cytosol do induce a response in yeast involving yeast native cell death pathways (see 69,70 for review), it appears that the permeabilization of mitochondrial membranes by itself interferes with essential processes, such as import of proteins into mitochondria. Reported impairment of the protein import pathway that delivers mitochondrial carriers into the inner mitochondrial membrane 22 is likely the result of the release of essential small Tim proteins 71 from the intermembrane space of permeabilized mitochondria. This conclusion is also supported by the reported release of small Tim proteins (Tim10 and Tim13) from yeast mitochondria treated with recombinant Bax 68.

## ANTIAPOPTOTIC PROTEINS BCL-2 AND BCL-XL

To understand how Bcl-2 family proteins regulate the release of the cytochrome *c*, it is necessary to uncover how antiapoptotic proteins of the family regulate the activity of Bax and Bak. The expression of antiapoptotic proteins Bcl-XL and Bcl-2 in wild type yeast has no obvious phenotype when these proteins are expressed individually, but it is able to completely inhibit the function of co-expressed proapoptotic proteins Bax or Bak 55. In cells, co-expressing Bax or Bak together with Bcl-XL or Bcl-2, no release of cytochrome *c* can be observed 5.

### How do antiapoptotic proteins inhibit Bax?

**Figure 3:**
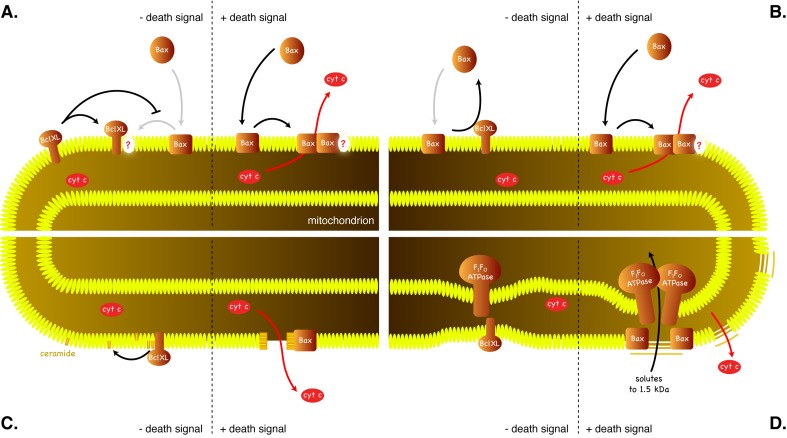
FIGURE 3: Models for inhibition of Bax by antiapoptotic proteins. **(A)** Competition for a mitochondrial binding site. In order to permeabilize mitochondrial outer membrane, Bax needs to bind to a specific binding site in the membrane. Bcl-XL binds to the same site with higher affinity, not allowing Bax to interact with the site. **(B)** Bax is constitutively targeted into mitochondrial membranes while Bcl-XL retrotranslocates Bax back to the cytosol. **(C)** Bax induces assembly of ceramide pores in the outer mitochondrial membrane and Bcl-XL disassembles these pores. **(D)** Bax interacts with F_1_F_O_-ATPase to induce permeability transition that results in swelling of mitochondria and ultimately the rupture of the outer mitochondrial membrane. Bcl-XL inhibits the opening of the permeability transition pore.

In order to address the nature of the interactions among the members of Bcl-2 family, the combinations of pro- and antiapoptotic proteins have been co-expressed in yeast. One of the typical features of Bcl-2 family proteins is their ability to heterodimerize 72,73. The significance of protein-protein interactions in inhibition of proapoptotic proteins by antiapoptotic proteins has been tested by using a mutant protein with mutations in BH3 domain, which is involved in Bcl-2 family protein heterodimerization. Introduction of the mutation (tyrosine in position 101 to lysine) into antiapoptotic protein Bcl-XL rendered the protein unable to heterodimerize with Bax but did not affect its ability to inhibit Bax 20,55,74, indicating that the ability to heterodimerize may not be required for the antiapoptotic function of Bcl-XL. When different amounts of Bax and Bcl-XL have been co-expressed, the amount of Bcl-XL protein required for yeast cell survival did not correlate with the amount of Bax expressed, rather a threshold amount of Bcl-XL was required to inhibit the activity of any amount of Bax tested 20. These observations further stress that mechanisms other than heterodimerization may play a crucial role in the antiapoptotic activity of Bcl-XL. As these mechanisms likely do not involve physical binding of Bcl-XL (and Bcl-2) to Bax (and Bak), a model, in which these two antagonistic proteins compete for a binding partner in the mitochondrial membrane (Figure 3A), was proposed 20.

Of the models, proposed for the inhibition of Bax by Bcl-XL, several others would be consistent with this observation in yeast. These include the model, in which Bcl-XL inhibits Bax by translocating it from mitochondria into the cytosol (Figure 3B) 75. According to this model, in living cells, Bax would constantly translocate from the cytosol to the mitochondrial membrane and would be retrotranslocated into the cytosol by Bcl-XL. When inhibited by BH3-only proteins, Bcl-XL would fail to retrotranslocate Bax, and Bax would form oligomers in the outer mitochondrial membrane.

Another model that would be consistent with the observations in yeast is a model, in which Bax induces the assembly of a lipid pore built from ceramide (Figure 3C). In this model molecules of ceramide align in a membrane with their hydrophobic tails oriented in parallel with the plane of the membrane into columns stabilized by intermolecule hydrogen bonds. These ceramide columns assemble into rings with hydrophilic heads lining a central pore 76,77. Such pores, with a diameter of 6-10 nm, are large enough to facilitate the diffusion of proteins of the size of the cytochrome *c* and their formation is observed when ceramide is added to phospholipid vesicles or to the isolated mitochondria. The addition of purified Bax to purified rat or yeast mitochondria stimulates the formation of ceramide pores 78,79, while the addition of Bcl-XL disassembles these pores and mitochondria isolated from yeast cells expressing Bcl-2 are not able to form ceramide pores 80. Moreover, involvement of ceramide in Bax-mediated permeabilization of the outer mitochondrial membrane would explain the Bax-suppressing effect of sphingomyelin synthase (mSMS1) 40 mentioned above.

Interesting observation from both mammalian and yeast cells points to yet another possible mechanism, by which Bcl-XL could antagonize the cell killing activity of Bax. Bcl-XL was found to stabilize the inner mitochondrial membrane potential and to physically interact with F_1_F_O_-ATPase 81. Authors suggest that Bcl-XL could prevent irregular fluctuations in the membrane potential by inhibiting the leak of ions across the inner mitochondrial membrane and that this leak may be mediated by F_1_F_O_-ATPase. Again, if F_1_F_O_-ATPase is a key constituent of the permeability transition pore, Bcl-XL would likely prevent its opening (Figure 3D).

### Non-apoptotic functions of Bcl-2 and Bcl-XL

Besides the inhibition of proapoptotic proteins, Bcl-2 and Bcl-XL have been reported to affect other cellular functions in yeast that are independent of the expression of other mammalian proteins. When Bcl-2 is expressed in yeast mutants lacking copper-zinc superoxide dismutase or manganese-containing superoxide dismutase due to the deletion of *SOD1* or *SOD2* genes, respectively, it significantly improves their stationary phase survival rate as well as their ability to form viable colonies when grown in hyperoxia 82. Expression of Bcl-2 and Bcl-XL also significantly increases the survival rate of yeast cells exposed to oxidative stress (treatment with hydrogen peroxide and menadion) and heat shock 83.

As it was observed that Bcl-XL may help mammalian cells facilitate a switch in metabolism when cells lose the ability to utilize glucose 84, a genetic screen to identify genes with analogous function in yeast was performed 85. In this screen, a library of yeast deletion mutants was searched for mutants, in which the expression of Bcl-XL improves growth when cells shift metabolism from fermentation to oxidative phosphorylation (diauxic shift). Thus identified genes include *TCM62*, a mitochondrial chaperone involved in assembly of the succinate dehydrogenase complex - a homologue of human prohibitin 86, and *SVF1*, an unknown factor required for diauxic shift and for survival under conditions of oxidative stress and cold stress 87.

Human Bcl-XL and Bcl-2 have also been found to increase the viability of yeast mutants with the deletion of *FIS1* gene in the presence of hydrogen peroxide or acetic acid 88. Fis1p is an outer mitochondrial membrane protein that is involved in mitochondrial fission 89. Besides its role in mitochondrial fission it has been found to be involved in the regulation of cell survival, as deletion mutants lacking Fis1p are more sensitive to acetic acid and hydrogen peroxide - two typical inducers of programmed cell death in yeast 88.

Taken together, these data show that antiapoptotic proteins Bcl-XL and Bcl-2 clearly have other functions in cells that are independent from their interactions with other Bcl-2 family members.

### Viral antiapoptotic proteins

Homologues of antiapoptotic proteins can be found in many viruses 90. These proteins, collectively referred to as vBcl-2 proteins, inhibit apoptosis in infected cells to enable virus to complete its replication cycle and to spread. Co-expression of these proteins with cellular Bcl-2 family proteins in yeast can provide a simple system, in which to study interactions between vBcl-2 and cellular Bcl-2 proteins. An example of such a protein studied in yeast is vBcl-2 encoded by M11 gene of Murine gammaherpesvirus 68. Co-expression of M11 with Bax or Bak in yeast saves cells from cell death induced by these proapoptotic proteins 91,92. In addition to the inhibition of apoptosis, in infected murine cells, M11 also inhibits autophagy that is dependent on the interaction of M11 with BH3 domain of Beclin-1, a protein involved in the regulation of autophagy 93. This effect is not observed in yeast 91. The inability of M11 to inhibit autophagy in yeast most likely reflects a poor homology between the yeast orthologue of Beclin - Atg6/Vps30 - and murine Beclin-1 in the region containing BH3 domain. Yeast cells expressing murine M11 (together with either Bax or Bak) thus represent an experimental system, in which Bax/Bak-inhibiting function of M11 can be separated from the effects of M11 on autophagy, which provides us with a further advantage.

### Yeast and screening for cancer drugs

Many types of cancer cells overexpress antiapoptotic proteins of the Bcl-2 family, which makes them resistant to apoptosis 94. Compounds specifically inhibiting relevant antiapoptotic proteins are therefore potentially interesting as anti-cancer drugs. A class of compounds that act as BH3-mimetics - they bind to BH3 peptide-binding groove of antiapoptotic proteins - includes for example ABT-263/Navitoclax 95, ABT-737 96 and GX15-070/Oblatoclax 97, some of which are in clinical trials for the cancer treatment. These molecules differ in their affinity to individual antiapoptotic proteins. Because for cancer treatment it is necessary that the drug specifically targets the antiapoptotic proteins that are overexpressed in a particular type of cancer cells, it would be desirable to have an easy assay for testing the activity and selectivity of candidate compounds. Yeast strains co-expressing Bax together with individual antiapoptotic proteins (Bcl-XL, Bcl-2, Bcl-w, A1 and Mcl-1) have been tested as a system, in which chemical compounds can be screened for their ability to inhibit antiapoptotic proteins 98. This system worked well with tested compounds (ABT-263/Navitoclax ABT-737) and as it is simple enough, it is likely that, if automatized, it can be used for high throughput screening.

## BH3-ONLY PROTEINS

As mentioned above, proteins of the Bcl-2 family homologous with Bcl-2 only in the BH3 domain constitute a distinct subfamily of this protein family. In living mammalian cells these proteins act as sentinels of cellular damage that respond to diverse cell death signals by activation of Bax and Bak 99 100. Individual BH3-only proteins differ in their intracellular localization in living cells and in the mechanism of their activation in response to death signal, which usually involves a posttranslational modification. For example, Bid is expressed as a 21 kDa protein localized in the cytosol. When cleaved by caspase, an active 15 kDa C-terminal fragment, tBid (truncated Bid), is produced. tBid then translocates to the mitochondria and activates the formation of a pore by Bax or Bak.

Several models have been proposed for the activation of Bax and Bak by BH3-only proteins. All these models generally reflect the fact that the BH3 domain is capable of binding into the hydrophobic groove in multidomain proteins consisting of BH1 and BH3 domains. BH3-only proteins are thus able to bind to both pro- and antiapoptotic multidomain proteins. In ‘direct activation’ model, activated BH3-only proteins bind to the inactive monomeric proapoptotic proteins Bax and Bak to induce their oligomerization and the formation of a pore in the mitochondrial membrane. ‘Indirect model’, on the other hand, assumes that active BH3-only proteins bind to antiapoptotic proteins like Bcl-XL and Bcl-2 to inhibit their antiapoptotic activity, which results in activation of Bax and Bak indirectly. Extensions of these models assume that some of the BH3-only proteins, called ‘activators‘ do directly activate Bax and Bak, while others, ‘sensitizers’, employ indirect mechanism 100 101.

Yeast co-expressing individual BH3-proteins together with pro- and antiapoptotic multidomain proteins does present a powerful tool to address the mechanisms of their interactions. Indeed, it has been demonstrated for several BH3-only proteins that their expression induces cell death in yeast in Bax- or Bak-dependent manner. Because the pathways that lead to the activation of BH3-only proteins in response to death signals in mammalian cells are absent in yeast, for some BH3-only proteins it may be necessary to express the modified versions of their genes, producing proteins that correspond to the processed active forms of these BH3-only proteins. For example, to study the activity of Bid, its truncated version (tBid) has to be expressed. Of many BH3-only proteins, the expression of several has been reported in yeast.

### Bid

As already mentioned, Bid (BH3 interacting-domain death agonist) is a cytosolic protein in mammalian cells that is activated by the cleavage with caspase 8, thus activating the mitochondrial apoptotic pathway when apoptosis is triggered by the extracellular ligands through a receptor pathway. Activated tBid interacts with mitochondrial membranes and activates Bax and Bak. Besides the activation of the pore assembly, tBid has been described to participate at mitochondrial cristae remodeling that likely mobilizes the pool of cytochrome *c*, most of which is trapped inside cristae unavailable for release by only permeabilization of the outer membrane 102.

The expression of tBid by itself in yeast does not induce any phenotype. When tBid is expressed together with multidomain proapoptotic proteins (Bax or Bak) and antiapoptotic proteins (Bcl-XL or Bcl-2), it triggers cell death 91 103. Cell killing potency of Bax or Bak is, however, not affected by the expression of tBid in the absence of Bcl-XL. As the phenotype depends on the presence of antiapoptotic proteins, the results of these experiments seem to indicate that tBid, in fact, inhibits antiapoptotic proteins. It is, however, important to note that in the experimental setting used in these experiments, Bax and Bak were active in the absence of other Bcl-2 proteins, which may have masked the direct effect of tBid on Bax and Bak.

Not detecting a direct activation is, however, in agreement with the results reported when the effect of tBid on both inactive and constitutively active Bax was analyzed 51. In this experiment, tBid was not able to induce killing in the presence of inactive Bax but did significantly enhance killing by active Bax, which may mean that tBid is not able to activate Bax directly but operates at the mitochondrial membrane in a way that contributes to the cell killing when active Bax is present. Yet in another paper, tBid is described to kill cells even in the absence of Bax and its cell killing activity is inhibited by the co-expression of Bcl-2 104.

### Bim

Bim (Bcl-2 interacting mediator) is the BH3-only protein involved in the apoptosis induced by stimuli involving antigen receptor ligation, Ca^2+^ flux, treatment with taxol or UV irradiation. In the absence of death signal, Bim is sequestered to the cytoskeleton by binding to the light chain of the dynein 105. Upon apoptotic stimulus, it is released from the complex and activates Bax or Bak to form pores in the mitochondrial membrane. Several isoforms of Bim exist due to the alternative splicing of the Bim transcript. The longest form, Bim_EL_, is produced from unspliced mRNA and other isoforms, including Bim_L_ and Bim_S_, are generated by the excision of introns 106. Individual Bim isoforms differ in the death-inducing potency, Bim_S_ being the most and Bim_EL_ being the least potent. The different potency of these proteins likely reflects the different regulation of their activity resulting from the absence of the domains of the protein that interact with other cellular proteins, for example the dynein binding domain is absent in Bim_S_.

The ability of purified Bim to induce the release of cytochrome *c* and to decrease the membrane potential of isolated mitochondria from both rat liver and yeast was reported 107. In these experiments the effect of Bim on yeast mitochondria depended on the presence of VDAC, as addition of Bim to mitochondria isolated from *POR1* deletion mutant had no effect. These data also indicated that the permeabilization of mitochondrial membranes by Bim would not require any other proteins of the Bcl-2 family, since these are not present in yeast mitochondria. Independence on other Bcl-2 family proteins, however, was not observed when Bim was expressed in yeast. When expressed in yeast, Bim is efficiently targeted and integrated into mitochondrial membranes even in the absence of other Bcl-2 family proteins, but when expressed alone, Bim has no effect on the cell viability and growth 108. The expression of either splice variant of Bim has been shown to induce cell dying only when co-expressed with Bax 109 110, or together with Bax and Bcl-XL 108. Deletion of *POR1* gene in the host strain has no effect on the phenotypes induced by the expression of Bim, indicating that its activity is VDAC-independent 108.

### Other BH3-only proteins

Other BH3-only proteins expressed in yeast include Bmf, Bad, Bik, Bnip3, Bnip3L, Noxa and Puma. Similarly to Bim, binding to the cytoskeleton regulates the activity of Bmf (Bcl-2 modifying factor). It is bound to the dynein light chain in the myosin V motor complex associated with actin microfilaments 111. The loss of cell adhesion or presence of toxins induces the detachment of Bmf and the activation of multidomain proapoptotic proteins. Bad (Bcl-2-associated death promoter) activates Bax and Bak in response to the absence of growth factors, e.g. in neurons of developing brain. In the presence of growth factors, phosphorylated Bad is sequestered by 14-3-3 proteins in the cytosol. When dephosphorylated, Bad translocates into the mitochondria 112. Bik (Bcl-2-interacting killer) is localized in the membrane of the endoplasmic reticulum (ER) and activates Bax by unknown mechanism without translocation into mitochondria 113. Bnip3 (Bcl-2/E1B-19K-interacting protein 3) proteins constitute a subclass of BH3-only proteins including Bnip3 and Bnip3L (Bnip3-like) proteins, expression of which is induced under conditions of cellular stress and proteins are subsequently translocated into the mitochondria, where they form homodimers and activate Bax and Bak 114. Noxa (latin for damage) 115 and Puma (p53 upregulated modulator of apoptosis) 116 are transcriptionally activated by p53 in response to DNA damage or other cellular stress.

The expression of these proteins in yeast has been reported. In these experiments a constitutively active version of Bax was used. When Bad, Puma, BNip3, Bnip3L and Noxa were co-expressed with Bax and Bcl-2, Bad and Puma were observed to induce cell death, while the expression of other three BH3-only proteins had no effect on cell growth 104. In other paper, Bmf, Noxa and Bik were reported to induce cell death when co-expressed with Bax or Bak and Bcl-XL or Bcl-2, but have no additional effect when co-expressed with Bax or Bak in the absence of antiapoptotic molecules 103. This, again, may indicate that these proteins act as inhibitors of antiapoptotic proteins and activate Bax or Bak indirectly. The activity of Puma was investigated also by co-expression with inactive Bax 53. In this experimental setting neither Puma nor Bax has any effect on yeast growth. When expressed together, however, cells die and death-inducing effect of these two proteins can be inhibited by co-expression of Bcl-XL. This would suggest that Puma activates Bax directly. Nevertheless, as the results for these BH3-only proteins are obtained using different expression systems and different versions of Bax, one has to be very careful when comparing the results and drawing conclusions.

## BXI1P/YBH3P - A YEAST PROTEIN IN QUESTION

As already mentioned, yeast-based screening of mouse genomic library led to the identification of BI-1 (Bax inhibitor 1), the first member of a novel class of Bax inhibitors residing in the endoplasmic reticulum and involved in the Ca^2+^ homeostasis 39 117. BI-1, also known as TMBIM6, together with at least five other highly conserved proteins - TMBIM1-5 - constitutes the Transmembrane Bax Inhibitor-1 Motif-containing (TMBIM) protein family. Because of their proposed inhibitory effect on apoptosis, these proteins seem to represent additional regulators of programmed cell death pathways, plainly not related to the well established apoptotic regulators belonging to the Bcl-2 protein family 118. Homologues of TMBIM family members were found in non-mammalian animals, plants, fungi, bacteria and viruses. The putative ortholog of mammalian TMBIM6/BI-1 with a high degree of sequence similarity to all human TMBIM family members has been also identified in *S. cerevisiae* 119 120, though its affiliation to TMBIM6 protein subfamily is ambiguous 121. Accordingly, it was suggested that TMBIM6/BI-1 homolog is absent in *S. cerevisiae* and yeast BI-1 is rather homologous to TMBIM4/GAAP protein subfamily 122. In any case, yeast TMBIM homolog, renamed to Bxi1p 119, exhibits the same cytoprotective effect against Bax-induced cell death as do all tested eukaryotic homologues of BI-1 when assayed in yeast. These BI-1 homologues from animals, plants and yeast provide cytoprotection also against other forms of cellular stress, including hydrogen peroxide-induced oxidative stress and heat shock. The pro-survival function of BI-1 homologues is dependent on the presence of the C-terminal protein domain, indicating evolutionarily conserved mechanism for their action 120. The mechanism of anti-programmed cell death function of Bxi1p was proposed, when its localization in the ER was revealed 119. Yeast strains lacking Bxi1 protein are more prone to ethanol- and glucose-induced programmed cell death. At the same time, they are more responsive to endoplasmic reticulum stress-inducing drugs, what is accompanied by the decreased unfolded protein response in *BXI1* deletion strain. Altogether, these results identified the endoplasmic reticulum-localized Bxi1 protein as an important link between the unfolded protein response and the programmed cell death in yeast 119. Further support for the involvement of the endoplasmic reticulum in the yeast cell death 123 is based on the observations that Bax directly activates the unfolded protein response by inducing splicing of *HAC1* gene mRNA and that the function of the transmembrane protein kinase Ire1p, that acts as the ER-resident ER stress sensor, is also involved in the Bax-induced cell death in yeast 124.

Interestingly, *in silico* search for homologues of BH3-only proteins in the yeast genome revealed a surprising hit: the gene identified and named *YBH3* (yeast BH-3 only protein) 125 was the same gene (YNL305C) as *BXI1*. The affiliation of Ybh3p/Bxi1p to the Bcl-2 protein family is based on the presence of the putative yeast BH3 domain sequence at the very C-terminus of the protein. Though BH3 motif in Ybh3 protein contains all three essential amino acids identifying the BH3 domain (L-4(X)-D-(D/E)), it also displays two unusual characteristics, unprecedented among classical BH3-only proteins: (i) yeast BH3 sequence is truncated because of its C-terminal localization, and (ii) it overlaps with the last transmembrane segment of the multimembrane-spanning protein with seven transmembrane domains, which by itself is another atypical feature for the classical BH3 domain-containing proteins 101. Despite these for BH3-only proteins unusual characteristics, Ybh3p was shown to interact with Bcl-XL and the BH3 domain within Ybh3p was found to be functional. The BH3 domain-dependent apoptosis in yeast is triggered by the translocation of the Ybh3 protein to the mitochondria and is mediated by the mitochondrial phosphate carrier Mir1p, as the deletion of *MIR1* gene abolished the translocation of Ybh3p to the mitochondria. A yeast strain with a deleted *MIR1* gene exhibits resistance to the induction of apoptosis and a prolonged lifespan, both replicative and chronological 125. Even though these data obviously contradict those reported for *BXI1*, there may be an agreement with the results of Bxi1 protein studies if Ybh3p would also enhance the unfolded protein response mediated by its functional BH3 motif, as it has been described that some of the BH3-only proteins provoke the signaling activity of endoplasmic reticulum stress sensor Ire1α 118,126.

Very recently, an insight into the function of Bxi1p/Ybh3p came from bacteria. A homolog of BI-1 (BsYetJ) was identified in *Bacillus subtilis* and its crystal structure was determined 121. The structure consists of seven transmembrane α-helices (7TMs) and reversible transitions between open and closed conformations are driven by changes in pH 121. A pair of aspartate residues engaged in hydrogen bonding is crucial for these conformational transitions associated with calcium leak across the membrane. Sequence alignments of bacterial BsYetJ and human TMBIMs showed that both aspartates, residing in TM6 and TM7, are conserved not only in hBI-1/TMBIM6 but also in human TMBIM1-5 proteins 121. Most importantly, these aspartates are fully conserved also in yeast Bxi1p/Ybh3p protein 120, strongly supporting the view that this is not an atypical BH3-only protein but rather a classical member of Transmembrane Bax Inhibitor-1 Motif-containing protein family with a role in calcium homeostasis maintenance and endoplasmic stress response pathways.

## CONCLUSIONS

Yeast is a traditional eukaryotic model organism and has been particularly valuable in elucidating processes involving mitochondria. In the last twenty years yeast has become a model for cell death. In this review, we tried to recapitulate the most important contributions to our understanding of the action of mammalian Bcl-2 family proteins that were generated using yeast model system. Although many questions have been successfully answered, there is still no consensus on basic questions, including how Bax permeabilizes mitochondrial membranes, how antiapoptotic proteins prevent the membrane permeabilization or how BH3-only proteins mobilize Bax and Bak, to name the most prominent ones. It seems reasonable to expect that yeast systems that have been developed and used to study proteins of the Bcl-2 family and mechanisms involved in their action will remain a valuable tool capable of providing crucial data on this topic in years to come.

## References

[1] Adams JM, Cory S (2007). The Bcl-2 apoptotic switch in cancer development and therapy.. Oncogene.

[2] Youle RJ, Strasser A (2008). The BCL-2 protein family: opposing activities that mediate cell death.. Nat Rev Mol Cell Biol.

[3] Sato T, Hanada M, Bodrug S, Irie S, Iwama N, Boise LH, Thompson CB, Golemis E, Fong L, Wang HG (1994). Interactions among members of the Bcl-2 protein family analyzed with a yeast two-hybrid system.. Proc Natl Acad Sci U S A.

[4] Zha H, Fisk HA, Yaffe MP, Mahajan N, Herman B, Reed JC (1996). Structure-function comparisons of the proapoptotic protein Bax in yeast and mammalian cells.. Mol Cell Biol.

[5] Manon S, Chaudhuri B, Guerin M (1997). Release of cytochrome c and decrease of cytochrome c oxidase in Bax-expressing yeast cells, and prevention of these effects by coexpression of Bcl-xL.. FEBS Lett.

[6] Ligr M, Madeo F, Frohlich E, Hilt W, Frohlich KU, Wolf DH (1998). Mammalian Bax triggers apoptotic changes in yeast.. FEBS Lett.

[7] Madeo F, Frohlich E, Frohlich KU (1997). A yeast mutant showing diagnostic markers of early and late apoptosis.. J Cell Biol.

[8] Carmona-Gutierrez D, Eisenberg T, Buttner S, Meisinger C, Kroemer G, Madeo F (2010). Apoptosis in yeast: triggers, pathways, subroutines.. Cell Death Differ.

[9] Pavlov EV, Priault M, Pietkiewicz D, Cheng EH, Antonsson B, Manon S, Korsmeyer SJ, Mannella CA, Kinnally KW (2001). A novel, high conductance channel of mitochondria linked to apoptosis in mammalian cells and Bax expression in yeast.. J Cell Biol.

[10] Antonsson B, Conti F, Ciavatta A, Montessuit S, Lewis S, Martinou I, Bernasconi L, Bernard A, Mermod JJ, Mazzei G, Maundrell K, Gambale F, Sadoul R, Martinou JC (1997). Inhibition of Bax channel-forming activity by Bcl-2.. Science.

[11] Antonsson B, Montessuit S, Lauper S, Eskes R, Martinou JC (2000). Bax oligomerization is required for channel-forming activity in liposomes and to trigger cytochrome c release from mitochondria.. Biochem J.

[12] Lewis S, Bethell SS, Patel S, Martinou JC, Antonsson B (1998). Purification and biochemical properties of soluble recombinant human Bax.. Protein Expr Purif.

[13] Saito M, Korsmeyer SJ, Schlesinger PH (2000). BAX-dependent transport of cytochrome c reconstituted in pure liposomes.. Nat Cell Biol.

[14] Azzolin L, von Stockum S, Basso E, Petronilli V, Forte MA, Bernardi P (2010). The mitochondrial permeability transition from yeast to mammals.. FEBS Lett.

[15] Marzo I, Brenner C, Zamzami N, Jurgensmeier JM, Susin SA, Vieira HL, Prevost MC, Xie Z, Matsuyama S, Reed JC, Kroemer G (1998). Bax and adenine nucleotide translocator cooperate in the mitochondrial control of apoptosis.. Science.

[16] Shimizu S, Ide T, Yanagida T, Tsujimoto Y (2000). Electrophysiological study of a novel large pore formed by Bax and the voltage-dependent anion channel that is permeable to cytochrome c.. J Biol Chem.

[17] Shimizu S, Narita M, Tsujimoto Y (1999). Bcl-2 family proteins regulate the release of apoptogenic cytochrome c by the mitochondrial channel VDAC.. Nature.

[18] Shimizu S, Shinohara Y, Tsujimoto Y (2000). Bax and Bcl-xL independently regulate apoptotic changes of yeast mitochondria that require VDAC but not adenine nucleotide translocator.. Oncogene.

[19] Gross A, Pilcher K, Blachly-Dyson E, Basso E, Jockel J, Bassik MC, Korsmeyer SJ, Forte M (2000). Biochemical and genetic analysis of the mitochondrial response of yeast to BAX and BCL-X(L).. Mol Cell Biol.

[20] Polcic P, Forte M (2003). Response of yeast to the regulated expression of proteins in the Bcl-2 family.. Biochem J.

[21] Priault M, Camougrand N, Chaudhuri B, Schaeffer J, Manon S (1999). Comparison of the effects of bax-expression in yeast under fermentative and respiratory conditions: investigation of the role of adenine nucleotides carrier and cytochrome c.. FEBS Lett.

[22] Kissova I, Polcic P, Kempna P, Zeman I, Sabova L, Kolarov J (2000). The cytotoxic action of Bax on yeast cells does not require mitochondrial ADP/ATP carrier but may be related to its import to the mitochondria.. FEBS Lett.

[23] Basso E, Fante L, Fowlkes J, Petronilli V, Forte MA, Bernardi P (2005). Properties of the permeability transition pore in mitochondria devoid of Cyclophilin D.. J Biol Chem.

[24] Nakagawa T, Shimizu S, Watanabe T, Yamaguchi O, Otsu K, Yamagata H, Inohara H, Kubo T, Tsujimoto Y (2005). Cyclophilin D-dependent mitochondrial permeability transition regulates some necrotic but not apoptotic cell death.. Nature.

[25] Schinzel AC, Takeuchi O, Huang Z, Fisher JK, Zhou Z, Rubens J, Hetz C, Danial NN, Moskowitz MA, Korsmeyer SJ (2005). Cyclophilin D is a component of mitochondrial permeability transition and mediates neuronal cell death after focal cerebral ischemia.. Proc Natl Acad Sci U S A.

[26] Matsuyama S, Xu Q, Velours J, Reed JC (1998). The Mitochondrial F0F1-ATPase proton pump is required for function of the proapoptotic protein Bax in yeast and mammalian cells.. Mol Cell.

[27] Giorgio V, von Stockum S, Antoniel M, Fabbro A, Fogolari F, Forte M, Glick GD, Petronilli V, Zoratti M, Szabo I, Lippe G, Bernardi P (2013). Dimers of mitochondrial ATP synthase form the permeability transition pore.. Proc Natl Acad Sci U S A.

[28] Carraro M, Giorgio V, Sileikyte J, Sartori G, Forte M, Lippe G, Zoratti M, Szabo I, Bernardi P (2014). Channel formation by yeast F-ATP synthase and the role of dimerization in the mitochondrial permeability transition.. J Biol Chem.

[29] Lutter M, Fang M, Luo X, Nishijima M, Xie X, Wang X (2000). Cardiolipin provides specificity for targeting of tBid to mitochondria.. Nat Cell Biol.

[30] Kuwana T, Mackey MR, Perkins G, Ellisman MH, Latterich M, Schneiter R, Green DR, Newmeyer DD (2002). Bid, Bax, and lipids cooperate to form supramolecular openings in the outer mitochondrial membrane.. Cell.

[31] Jiang F, Rizavi HS, Greenberg ML (1997). Cardiolipin is not essential for the growth of Saccharomyces cerevisiae on fermentable or non-fermentable carbon sources.. Mol Microbiol.

[32] Chang SC, Heacock PN, Clancey CJ, Dowhan W (1998). The PEL1 gene (renamed PGS1) encodes the phosphatidylglycero-phosphate synthase of Saccharomyces cerevisiae.. J Biol Chem.

[33] Zhang M, Mileykovskaya E, Dowhan W (2002). Gluing the respiratory chain together. Cardiolipin is required for supercomplex formation in the inner mitochondrial membrane.. J Biol Chem.

[34] Janitor M, Subik J (1993). Molecular cloning of the PEL1 gene of Saccharomyces cerevisiae that is essential for the viability of petite mutants.. Curr Genet.

[35] Iverson SL, Enoksson M, Gogvadze V, Ott M, Orrenius S (2004). Cardiolipin is not required for Bax-mediated cytochrome c release from yeast mitochondria.. J Biol Chem.

[36] Polcic P, Su X, Fowlkes J, Blachly-Dyson E, Dowhan W, Forte M (2005). Cardiolipin and phosphatidylglycerol are not required for the in vivo action of Bcl-2 family proteins.. Cell Death Differ.

[37] Gonzalvez F, Bessoule JJ, Rocchiccioli F, Manon S, Petit PX (2005). Role of cardiolipin on tBid and tBid/Bax synergistic effects on yeast mitochondria.. Cell Death Differ.

[38] Gonzalvez F, Pariselli F, Dupaigne P, Budihardjo I, Lutter M, Antonsson B, Diolez P, Manon S, Martinou JC, Goubern M, Wang X, Bernard S, Petit PX (2005). tBid interaction with cardiolipin primarily orchestrates mitochondrial dysfunctions and subsequently activates Bax and Bak.. Cell Death Differ.

[39] Xu Q, Reed JC (1998). Bax inhibitor-1, a mammalian apoptosis suppressor identified by functional screening in yeast.. Mol Cell.

[40] Yang Z, Khoury C, Jean-Baptiste G, Greenwood MT (2006). Identification of mouse sphingomyelin synthase 1 as a suppressor of Bax-mediated cell death in yeast.. FEMS Yeast Res.

[41] Huitema K, van den Dikkenberg J, Brouwers JF, Holthuis JC (2004). Identification of a family of animal sphingomyelin synthases.. EMBO J.

[42] Kampranis SC, Damianova R, Atallah M, Toby G, Kondi G, Tsichlis PN, Makris AM (2000). A novel plant glutathione S-transferase/peroxidase suppresses Bax lethality in yeast.. J Biol Chem.

[43] Chen S, Vaghchhipawala Z, Li W, Asard H, Dickman MB (2004). Tomato phospholipid hydroperoxide glutathione peroxidase inhibits cell death induced by Bax and oxidative stresses in yeast and plants.. Plant Physiol.

[44] Dimitrova I, Toby GG, Tili E, Strich R, Kampranis SC, Makris AM (2004). Expression of Bax in yeast affects not only the mitochondria but also vacuolar integrity and intracellular protein traffic.. FEBS Lett.

[45] Bounhar Y, Zhang Y, Goodyer CG, LeBlanc A (2001). Prion protein protects human neurons against Bax-mediated apoptosis.. J Biol Chem.

[46] Prusiner SB, Scott MR, DeArmond SJ, Cohen FE (1998). Prion protein biology.. Cell.

[47] Li A, Harris DA (2005). Mammalian Prion Protein Suppresses Bax-induced Cell Death in Yeast.. J Biol Chem.

[48] Bounhar Y, Mann KK, Roucou X, LeBlanc AC (2006). Prion protein prevents Bax-mediated cell death in the absence of other Bcl-2 family members in Saccharomyces cerevisiae.. FEMS Yeast Res.

[49] Jodoin J, Laroche-Pierre S, Goodyer CG, LeBlanc AC (2007). Defective retrotranslocation causes loss of anti-Bax function in human familial prion protein mutants.. J Neurosci.

[50] Jodoin J, Misiewicz M, Makhijani P, Giannopoulos PN, Hammond J, Goodyer CG, LeBlanc AC (2009). Loss of anti-Bax function in Gerstmann-Straussler-Scheinker syndrome-associated prion protein mutants.. PLoS One.

[51] Priault M, Cartron PF, Camougrand N, Antonsson B, Vallette FM, Manon S (2003). Investigation of the role of the C-terminus of Bax and of tc-Bid on Bax interaction with yeast mitochondria.. Cell Death Differ.

[52] Arokium H, Camougrand N, Vallette FM, Manon S (2004). Studies of the interaction of substituted mutants of BAX with yeast mitochondria reveal that the C-terminal hydrophobic alpha-helix is a second ART sequence and plays a role in the interaction with anti-apoptotic BCL-xL.. J Biol Chem.

[53] Gallenne T, Gautier F, Oliver L, Hervouet E, Noel B, Hickman JA, Geneste O, Cartron PF, Vallette FM, Manon S, Juin P (2009). Bax activation by the BH3-only protein Puma promotes cell dependence on antiapoptotic Bcl-2 family members.. J Cell Biol.

[54] Renault TT, Manon S (2011). Bax: Addressed to kill.. Biochimie.

[55] Tao W, Kurschner C, Morgan JI (1997). Modulation of cell death in yeast by the Bcl-2 family of proteins.. J Biol Chem.

[56] Greenhalf W, Stephan C, Chaudhuri B (1996). Role of mitochondria and C-terminal membrane anchor of Bcl-2 in Bax induced growth arrest and mortality in Saccharomyces cerevisiae.. FEBS Lett.

[57] Suzuki M, Youle RJ, Tjandra N (2000). Structure of Bax: coregulation of dimer formation and intracellular localization.. Cell.

[58] Muchmore SW, Sattler M, Liang H, Meadows RP, Harlan JE, Yoon HS, Nettesheim D, Chang BS, Thompson CB, Wong SL, Ng SL, Fesik SW (1996). X-ray and NMR structure of human Bcl-xL, an inhibitor of programmed cell death.. Nature.

[59] Oliver L, Priault M, Tremblais K, LeCabellec M, Meflah K, Manon S, Vallette FM (2000). The substitution of the C-terminus of bax by that of bcl-xL does not affect its subcellular localization but abrogates its pro-apoptotic properties.. FEBS Lett.

[60] Gardai SJ, Hildeman DA, Frankel SK, Whitlock BB, Frasch SC, Borregaard N, Marrack P, Bratton DL, Henson PM (2004). Phosphorylation of Bax Ser184 by Akt regulates its activity and apoptosis in neutrophils.. J Biol Chem.

[61] Jin KL, Graham SH, Mao XO, He X, Nagayama T, Simon RP, Greenberg DA (2001). Bax kappa, a novel Bax splice variant from ischemic rat brain lacking an ART domain, promotes neuronal cell death.. J Neurochem.

[62] Cartron PF, Oliver L, Martin S, Moreau C, LeCabellec MT, Jezequel P, Meflah K, Vallette FM (2002). The expression of a new variant of the pro-apoptotic molecule Bax, Baxpsi, is correlated with an increased survival of glioblastoma multiforme patients.. Hum Mol Genet.

[63] Cartron PF, Priault M, Oliver L, Meflah K, Manon S, Vallette FM (2003). The N-terminal end of Bax contains a mitochondrial-targeting signal.. J Biol Chem.

[64] Arokium H, Ouerfelli H, Velours G, Camougrand N, Vallette FM, Manon S (2007). Substitutions of potentially phosphorylatable serine residues of Bax reveal how they may regulate its interaction with mitochondria.. J Biol Chem.

[65] Bellot G, Cartron PF, Er E, Oliver L, Juin P, Armstrong LC, Bornstein P, Mihara K, Manon S, Vallette FM (2007). TOM22, a core component of the mitochondria outer membrane protein translocation pore, is a mitochondrial receptor for the proapoptotic protein Bax.. Cell Death Differ.

[66] Ott M, Norberg E, Walter KM, Schreiner P, Kemper C, Rapaport D, Zhivotovsky B, Orrenius S (2007). The mitochondrial TOM complex is required for tBid/Bax-induced cytochrome c release.. J Biol Chem.

[67] Renault TT, Grandier-Vazeille X, Arokium H, Velours G, Camougrand N, Priault M, Teijido O, Dejean LM, Manon S (2012). The cytosolic domain of human Tom22 modulates human Bax mitochondrial translocation and conformation in yeast.. FEBS Lett.

[68] Sanjuan Szklarz LK, Kozjak-Pavlovic V, Vogtle FN, Chacinska A, Milenkovic D, Vogel S, Durr M, Westermann B, Guiard B, Martinou JC, Borner C, Pfanner N, Meisinger C (2007). Preprotein transport machineries of yeast mitochondrial outer membrane are not required for Bax-induced release of intermembrane space proteins.. J Mol Biol.

[69] Khoury CM, Greenwood MT (2008). The pleiotropic effects of heterologous Bax expression in yeast.. Biochim Biophys Acta.

[70] Pereira C, Silva RD, Saraiva L, Johansson B, Sousa MJ, Corte-Real M (2008). Mitochondria-dependent apoptosis in yeast.. Biochim Biophys Acta.

[71] Pfanner N (1998). Mitochondrial import: crossing the aqueous intermembrane space.. Curr Biol.

[72] Yin XM, Oltvai ZN, Korsmeyer SJ (1994). BH1 and BH2 domains of Bcl-2 are required for inhibition of apoptosis and heterodimerization with Bax.. Nature.

[73] Sedlak TW, Oltvai ZN, Yang E, Wang K, Boise LH, Thompson CB, Korsmeyer SJ (1995). Multiple Bcl-2 family members demonstrate selective dimerizations with Bax.. Proc Natl Acad Sci U S A.

[74] Minn AJ, Kettlun CS, Liang H, Kelekar A, Vander Heiden MG, Chang BS, Fesik SW, Fill M, Thompson CB (1999). Bcl-xL regulates apoptosis by heterodimerization-dependent and -independent mechanisms.. EMBO J.

[75] Edlich F, Banerjee S, Suzuki M, Cleland MM, Arnoult D, Wang C, Neutzner A, Tjandra N, Youle RJ (2011). Bcl-x(L) retrotranslocates Bax from the mitochondria into the cytosol.. Cell.

[76] Siskind LJ (2005). Mitochondrial ceramide and the induction of apoptosis.. J Bioenerg Biomembr.

[77] Siskind LJ, Colombini M (2000). The lipids C2- and C16-ceramide form large stable channels. Implications for apoptosis.. J Biol Chem.

[78] Ganesan V, Colombini M (2010). Regulation of ceramide channels by Bcl-2 family proteins.. FEBS Lett.

[79] Ganesan V, Perera MN, Colombini D, Datskovskiy D, Chadha K, Colombini M (2010). Ceramide and activated Bax act synergistically to permeabilize the mitochondrial outer membrane.. Apoptosis.

[80] Siskind LJ, Feinstein L, Yu T, Davis JS, Jones D, Choi J, Zuckerman JE, Tan W, Hill RB, Hardwick JM, Colombini M (2008). Anti-apoptotic Bcl-2 Family Proteins Disassemble Ceramide Channels.. J Biol Chem.

[81] Chen YB, Aon MA, Hsu YT, Soane L, Teng X, McCaffery JM, Cheng WC, Qi B, Li H, Alavian KN, Dayhoff-Brannigan M, Zou S, Pineda FJ, O'Rourke B, Ko YH, Pedersen PL, Kaczmarek LK, Jonas EA, Hardwick JM (2011). Bcl-xL regulates mitochondrial energetics by stabilizing the inner membrane potential.. J Cell Biol.

[82] Longo VD, Ellerby LM, Bredesen DE, Valentine JS, Gralla EB (1997). Human Bcl-2 reverses survival defects in yeast lacking superoxide dismutase and delays death of wild-type yeast.. J Cell Biol.

[83] Chen SR, Dunigan DD, Dickman MB (2003). Bcl-2 family members inhibit oxidative stress-induced programmed cell death in Saccharomyces cerevisiae.. Free Radic Biol Med.

[84] Vander Heiden MG, Chandel NS, Li XX, Schumacker PT, Colombini M, Thompson CB (2000). Outer mitochondrial membrane permeability can regulate coupled respiration and cell survival.. Proc Natl Acad Sci U S A.

[85] Vander Heiden MG, Choy JS, VanderWeele DJ, Brace JL, Harris MH, Bauer DE, Prange B, Kron SJ, Thompson CB, Rudin CM (2002). Bcl-x(L) complements Saccharomyces cerevisiae genes that facilitate the switch from glycolytic to oxidative metabolism.. J Biol Chem.

[86] Dibrov E, Fu S, Lemire BD (1998). The Saccharomyces cerevisiae TCM62 gene encodes a chaperone necessary for the assembly of the mitochondrial succinate dehydrogenase (complex II).. J Biol Chem.

[87] Brace JL, Vanderweele DJ, Rudin CM (2005). Svf1 inhibits reactive oxygen species generation and promotes survival under conditions of oxidative stress in Saccharomyces cerevisiae.. Yeast.

[88] Fannjiang Y, Cheng WC, Lee SJ, Qi B, Pevsner J, McCaffery JM, Hill RB, Basanez G, Hardwick JM (2004). Mitochondrial fission proteins regulate programmed cell death in yeast.. Genes Dev.

[89] Mozdy AD, McCaffery JM, Shaw JM (2000). Dnm1p GTPase-mediated mitochondrial fission is a multi-step process requiring the novel integral membrane component Fis1p.. J Cell Biol.

[90] Cuconati A, White E (2002). Viral homologs of BCL-2: role of apoptosis in the regulation of virus infection.. Genes Dev.

[91] Juhasova B, Bhatia-Kissova I, Polcicova K, Mentel M, Forte M, Polcic P (2011). Reconstitution of interactions of Murine gammaherpesvirus 68 M11 with Bcl-2 family proteins in yeast.. Biochem Biophys Res Commun.

[92] Loh J, Huang Q, Petros AM, Nettesheim D, van Dyk LF, Labrada L, Speck SH, Levine B, Olejniczak ET, Virgin HWt (2005). A surface groove essential for viral Bcl-2 function during chronic infection in vivo.. PLoS Pathog.

[93] Sinha S, Colbert CL, Becker N, Wei Y, Levine B (2008). Molecular basis of the regulation of Beclin 1-dependent autophagy by the gamma-herpesvirus 68 Bcl-2 homolog M11.. Autophagy.

[94] Kelly PN, Strasser A (2011). The role of Bcl-2 and its pro-survival relatives in tumourigenesis and cancer therapy.. Cell Death Differ.

[95] Park CM, Bruncko M, Adickes J, Bauch J, Ding H, Kunzer A, Marsh KC, Nimmer P, Shoemaker AR, Song X, Tahir SK, Tse C, Wang X, Wendt MD, Yang X, Zhang H, Fesik SW, Rosenberg SH, Elmore SW (2008). Discovery of an orally bioavailable small molecule inhibitor of prosurvival B-cell lymphoma 2 proteins.. J Med Chem.

[96] Oltersdorf T, Elmore SW, Shoemaker AR, Armstrong RC, Augeri DJ, Belli BA, Bruncko M, Deckwerth TL, Dinges J, Hajduk PJ, Joseph MK, Kitada S, Korsmeyer SJ, Kunzer AR, Letai A, Li C, Mitten MJ, Nettesheim DG, Ng S, Nimmer PM, O'Connor JM, Oleksijew A, Petros AM, Reed JC, Shen W, Tahir SK, Thompson CB, Tomaselli KJ, Wang B, Wendt MD (2005). An inhibitor of Bcl-2 family proteins induces regression of solid tumours.. Nature.

[97] Campas C, Cosialls AM, Barragan M, Iglesias-Serret D, Santidrian AF, Coll-Mulet L, de Frias M, Domingo A, Pons G, Gil J (2006). Bcl-2 inhibitors induce apoptosis in chronic lymphocytic leukemia cells.. Exp Hematol.

[98] Beaumont TE, Shekhar TM, Kaur L, Pantaki-Eimany D, Kvansakul M, Hawkins CJ (2013). Yeast techniques for modeling drugs targeting Bcl-2 and caspase family members.. Cell Death Dis.

[99] Shibue T, Taniguchi T (2006). BH3-only proteins: integrated control point of apoptosis.. Int J Cancer.

[100] Willis SN, Adams JM (2005). Life in the balance: how BH3-only proteins induce apoptosis.. Curr Opin Cell Biol.

[101] Aouacheria A, Rech de Laval V, Combet C, Hardwick JM (2012). Evolution of Bcl-2 homology motifs: homology versus homoplasy.. Trends Cell Biol.

[102] Scorrano L, Ashiya M, Buttle K, Weiler S, Oakes SA, Mannella CA, Korsmeyer SJ (2002). A distinct pathway remodels mitochondrial cristae and mobilizes cytochrome c during apoptosis.. Dev Cell.

[103] Gerecova G, Kopanicova J, Jaka P, Behalova L, Juhasova B, Bhatia-Kissova I, Forte M, Polcic P, Mentel M (2013). BH3-only proteins Noxa, Bik, Bmf, and Bid activate Bax and Bak indirectly when studied in yeast model.. FEMS Yeast Res.

[104] Guscetti F, Nath N, Denko N (2005). Functional characterization of human proapoptotic molecules in yeast S. cerevisiae.. Faseb J.

[105] Puthalakath H, Huang DC, O'Reilly LA, King SM, Strasser A (1999). The proapoptotic activity of the Bcl-2 family member Bim is regulated by interaction with the dynein motor complex.. Mol Cell.

[106] O'Connor L, Strasser A, O'Reilly LA, Hausmann G, Adams JM, Cory S, Huang DC (1998). Bim: a novel member of the Bcl-2 family that promotes apoptosis.. EMBO J.

[107] Sugiyama T, Shimizu S, Matsuoka Y, Yoneda Y, Tsujimoto Y (2002). Activation of mitochondrial voltage-dependent anion channel by apro-apoptotic BH3-only protein Bim.. Oncogene.

[108] Juhasova B, Mentel M, Bhatia-Kissova I, Zeman I, Kolarov J, Forte M, Polcic P (2011). BH3-only protein Bim inhibits activity of antiapoptotic members of Bcl-2 family when expressed in yeast.. FEBS Lett.

[109] Weber A, Paschen SA, Heger K, Wilfling F, Frankenberg T, Bauerschmitt H, Seiffert BM, Kirschnek S, Wagner H, Hacker G (2007). BimS-induced apoptosis requires mitochondrial localization but not interaction with anti-apoptotic Bcl-2 proteins.. J Cell Biol.

[110] Wilfling F, Weber A, Potthoff S, Vogtle FN, Meisinger C, Paschen SA, Hacker G (2012). BH3-only proteins are tail-anchored in the outer mitochondrial membrane and can initiate the activation of Bax.. Cell Death Differ.

[111] Puthalakath H, Villunger A, O'Reilly LA, Beaumont JG, Coultas L, Cheney RE, Huang DC, Strasser A (2001). Bmf: a proapoptotic BH3-only protein regulated by interaction with the myosin V actin motor complex, activated by anoikis.. Science.

[112] Zha J, Harada H, Yang E, Jockel J, Korsmeyer SJ (1996). Serine phosphorylation of death agonist BAD in response to survival factor results in binding to 14-3-3 not BCL-X(L).. Cell.

[113] Germain M, Mathai JP, Shore GC (2002). BH-3-only BIK functions at the endoplasmic reticulum to stimulate cytochrome c release from mitochondria.. J Biol Chem.

[114] Chinnadurai G, Vijayalingam S, Gibson SB (2008). BNIP3 subfamily BH3-only proteins: mitochondrial stress sensors in normal and pathological functions.. Oncogene.

[115] Oda E, Ohki R, Murasawa H, Nemoto J, Shibue T, Yamashita T, Tokino T, Taniguchi T, Tanaka N (2000). Noxa, a BH3-only member of the Bcl-2 family and candidate mediator of p53-induced apoptosis.. Science.

[116] Nakano K, Vousden KH (2001). PUMA, a novel proapoptotic gene, is induced by p53.. Mol Cell.

[117] Xu C, Xu W, Palmer AE, Reed JC (2008). BI-1 regulates endoplasmic reticulum Ca2+ homeostasis downstream of Bcl-2 family proteins.. J Biol Chem.

[118] Rojas-Rivera D, Hetz C (2014). TMBIM protein family: ancestral regulators of cell death.. Oncogene.

[119] Cebulski J, Malouin J, Pinches N, Cascio V, Austriaco N (2011). Yeast Bax inhibitor, Bxi1p, is an ER-localized protein that links the unfolded protein response and programmed cell death in Saccharomyces cerevisiae.. PLoS One.

[120] Chae HJ, Ke N, Kim HR, Chen S, Godzik A, Dickman M, Reed JC (2003). Evolutionarily conserved cytoprotection provided by Bax Inhibitor-1 homologs from animals, plants, and yeast.. Gene.

[121] Chang Y, Bruni R, Kloss B, Assur Z, Kloppmann E, Rost B, Hendrickson WA, Liu Q (2014). Structural basis for a pH-sensitive calcium leak across membranes.. Science.

[122] Henke N, Lisak DA, Schneider L, Habicht J, Pergande M, Methner A (2011). The ancient cell death suppressor BAX inhibitor-1.. Cell Calcium.

[123] Austriaco N (2012). Endoplasmic reticulum involvement in yeast cell death.. Front Oncol.

[124] Cakir B (2012). Bax induces activation of the unfolded protein response by inducing HAC1 mRNA splicing in Saccharomyces cerevisiae.. Yeast.

[125] Buttner S, Ruli D, Vogtle FN, Galluzzi L, Moitzi B, Eisenberg T, Kepp O, Habernig L, Carmona-Gutierrez D, Rockenfeller P, Laun P, Breitenbach M, Khoury C, Frohlich KU, Rechberger G, Meisinger C, Kroemer G, Madeo F (2011). A yeast BH3-only protein mediates the mitochondrial pathway of apoptosis.. EMBO J.

[126] Rodriguez DA, Zamorano S, Lisbona F, Rojas-Rivera D, Urra H, Cubillos-Ruiz JR, Armisen R, Henriquez DR, Cheng EH, Letek M, Vaisar T, Irrazabal T, Gonzalez-Billault C, Letai A, Pimentel-Muinos FX, Kroemer G, Hetz C (2012). BH3-only proteins are part of a regulatory network that control the sustained signalling of the unfolded protein response sensor IRE1alpha.. EMBO J.

